# Cathepsin B, D and S as Potential Biomarkers of Brain Glioma Malignancy

**DOI:** 10.3390/jcm11226763

**Published:** 2022-11-15

**Authors:** Lukasz Oldak, Patrycja Milewska, Sylwia Chludzinska-Kasperuk, Kamil Grubczak, Joanna Reszec, Ewa Gorodkiewicz

**Affiliations:** 1 Bioanalysis Laboratory, Faculty of Chemistry, University of Bialystok, Ciolkowskiego 1K, 15-245 Bialystok, Poland; 2 Doctoral School of Exact and Natural Science, Faculty of Chemistry, University of Bialystok, Ciolkowskiego 1K, 15-245 Bialystok, Poland; 3 Biobank, Medical University of Bialystok, Waszyngtona 13, 15-269 Bialystok, Poland; 4 Department of Regenerative Medicine and Immune Regulation, Medical Univeristy of Bialystok, Waszyngtona 13, 15-269 Bialystok, Poland; 5 Department of Medical Pathology, Medical University of Bialystok, Waszyngtona 13, 15-269 Bialystok, Poland

**Keywords:** glioma, cathepsin B, cathepsin D, cathepsin S, cathepsin quantification, statistically analysis

## Abstract

Brain gliomas constitute the vast majority of malignant tumors of the nervous system. There is still a lack of fast, reliable and non-invasive methods of diagnostics. Our work focuses on the quantification of cathepsin B, D and S in glioma. The research was conducted with the use of SPRi biosensors sensitive to individual cathepsins. Changes in the quantity of selected cathepsins (cathepsins B, D and S), depending on the advancement of glioma and the presence or absence of important features or comorbidities in the selected patient, were examined. The results were statistically analyzed and interpreted based on the available clinical description. Statistical significance was observed in the difference in the concentration of the studied cathepsins, mainly between the groups Control and G3/G4 and G1/G2 and G3/G4. The strength of the correlation between the concentrations of individual cathepsins and the age of the patient and the size of the tumor, as well as the correlation between individual proteins, was investigated. The influence of IDH 1/2 status on the concentration of determined cathepsins was investigated and ROC analysis was performed. As a result of our research, we have developed a method for the diagnosis of brain glioma that allows us to distinguish grades G1/G2 from G3/G4 and the control group from G3/G4. We found an average positive correlation between the concentrations of the proteins tested and the age of the patient and a high positive correlation between the cathepsins tested. Comparative analysis of the effect of the presence of IDH 1/2 mutations on the number of proteins tested allowed us to demonstrate that the cathepsins assayed can be independent markers.

## 1. Introduction

Gliomas account for 80% of all malignant brain tumors of a primary nature. From a histopathological point of view, the current WHO classification and EANO (European Association of Neuro-Oncology) guidelines of 2021 divides gliomas into circumscribed or diffuse [1]. Each of them is characterized by different potential for tumor growth, recurrence of the disease, and possibility of complete cure [2,3]. The mildest grade (1) includes pilocytic astrocytomas. These are usually very slow-growing tumors. Complete resection leads to the best prognosis for a complete cure. Among diffuse gliomas, we distinguish grade 2 and 3 astrocytomas and oligodendrogliomas. The most aggressive are grade 4 astrocytomas and IDH-wild-type glioblastoma. They are also characterized by the shortest patient survival time, ranging from 14.5 to 16.6 months (median value). Nevertheless, the diagnosis and resection of the tumor is very difficult, especially in the lower grades of malignancy (2 and 3), due to the diffuse nature of these cancerous tumors. Very often, therefore, even after surgical intervention, tumors of the second and third grade are followed by rapid recurrence of the disease, but usually with a higher degree of malignancy, this being due to the presence of microscopic foci of cancer cells that were not removed during resection [4,5].

Currently, one of the greatest problems in the diagnosis of glioma is the lack of quick and effective strategies. The methods currently used are based mainly on neuroimaging methods. However, such tests are performed too late—usually at a time when the disease is already very advanced [6,7]. Late diagnosis occurs due to the slow process of spreading. Current brain structures can adapt to the deformation caused by the tumor mass. Therefore, clinical symptoms in patients appear late—only at the advanced disease stage. Other obstacles to imaging methods are antiangiogenic drugs and chemotherapy, because they falsify the results of neuroimaging, which makes correct diagnosis much more difficult [5].

Cathepsins are essential proteins in the human body, due to their role in the catalysis of protein degradation. This process is aimed at maintaining normal levels of amino acids. Disorders of the physiological activity of cathepsins are associated with various types of pathological processes. Cathepsin B (Cath B, most widely considered and discussed in the case of cancer) and S (Cath S) belong to the family of cysteine cathepsins, while cathepsin D (Cath D) is aspartic. Especially dangerous is the uncontrolled activity of cysteine cathepsins. It is mainly this family that leads to cancer. The extracellular environment of cancer cells creates an ideal place for most cathepsins, which are usually located outside the cancer cells, to function. These proteases most often exhibit their maximum catalytic activity in an acidic environment. They can activate growth factors and metalloproteinases (MMPs) and degrade the components of the extracellular matrix themselves. On examining the topic further, we learn that Cath B, by activating MMPs, contributes to the separation of cells, which in turn leads to initiation of the process of cellular migration [8,9]. In addition, it is responsible for the growth of cancer cells and their survival. Cath B is involved in the activation of signaling pathways responsible for starting the angiogenesis process. This causes MMP inhibitors to be deactivated. Previous studies have shown significantly higher Cath B levels for the most malignant and aggressive grade of glioma, glioblastoma multiforme, than for the lower grades of tumor malignancy. It was also found that the level of Cath B shows a significant correlation with the degree of invasiveness of glioma cells. A selective inhibitor against Cath B (CA074) reduces the invasiveness of cancer cells [10]. Cath B, as it undergoes very strong overexpression in the case of stage IV astrocytoma (GBM), is considered a predictor of the survival of a patient with the diagnosed disease, because the level of expression of this protease is significantly correlated with shorter survival of patients [11].

In gliomas, an increased level of Cath D expression has been observed—as in the case of cathepsin B—in grades characterized by a higher degree of malignancy. A significant positive correlation was also observed between Cath B and Cath D levels in blood serum and the invasive nature of the cancer. This is probably related to the high proteolytic activity of these cathepsins [12,13,14].

The role of Cath D in tumor progression has been demonstrated. It participates in the processes of cell proliferation, angiogenesis and apoptosis. Since Cath D is associated with the resistance of cancer cells to treatment, including those of liver, pancreatic and ovarian cancers, it has been concluded that this protease is one of the key lysosome proteins responsible for autophagy. Thus, high Cath D expression also increases the level of autophagy [15]. Cath D has one more key role: it can activate Cath B. In addition, in the brain it is responsible for specific cleavage and processing of brain proteins (e.g., myelin), it activates cysteine protease inhibitors, and it is also responsible for the conversion of procollagen to collagen and the degradation of ECM [16].

Cathepsin S can also degrade the basement membrane, and above all, proteins such as laminins, collagens and elastin and chondroitin sulfate proteoglycans. The increase in Cath S secretion is caused by inflammatory mediators and growth factors, including the basic fibroblast growth factor (bFGF). It is stable even at neutral pH; the optimal range for maximum catalytic activity is pH = 5.0–7.5, which entails an even greater increase in cancer invasiveness, outside the peri-cancer environment, where conditions are mainly acidic, very conducive to the activity of Cath B and D. Studies have shown that Cath S is expressed in astrocytomas, while healthy cells did not show an increase in the expression of this protease. However, as in the case of the previously described cathepsins, the highest level of Cath S activity was measured in tissue homogenates with a confirmed grade IV astrocytoma [17].

Surface Plasmon Resonance (SPR) is an optical technique used to study the kinetics of bonds between biomolecules, and for quantification. The technique is based on the measurement of changes in the refractive index during adsorption on the metal surface, for example of biomolecules. SPR works very well with appropriate biosensors, which are characterized by a simple structure and the lack of a need to use labels [18,19]. There are several varieties of SPR, including SPRi (Surface Plasmon Resonance imaging). With the use of SPRi, potential biomarkers such as HE4 [20] and UCH-L1 [21] have been successfully determined using standard gold chips, and modified, so-called bimetallic chips with dusted gold and silver in appropriate proportions have also been used for quantitative determinations, for example of Cath D [22] and Cath S [23].

The aim of our work was to develop a new method supporting the diagnosis of brain glioma, which uses SPRi biosensors sensitive to Cath B, Cath D and Cath S. We examined the effect of glioma grade on the concentration of individual proteins in the blood plasma. We also calculated the influence of the patient’s age and the size of the neoplastic tumor on the amounts of cathepsins determined. Moreover, we assessed the strength of the correlation between the studied proteins. We also checked whether the presence of neoplastic diseases in the patient’s family may increase the risk of developing brain glioma and analyzed how the presence of other comorbidities affects the malignancy of brain glioma. The measure in both analyzed cases was an increase in Cath B, Cath D and Cath S concentrations compared to the control group. We checked whether the proposed biomarkers could serve as independent prognostic factors of glioma malignancy by examining the effect of IDH 1/2 status on the quantified cathepsins. To assess the diagnostic usefulness of the cathepsins in the case of brain glioma, we performed ROC analysis. The control group consisted of heavy smokers who did not show symptoms characteristic of brain glioma. It was selected to correspond to the group of patients in terms of age.

## 2. Materials and Methods

Cathepsin B from human liver, cysteamine hydrochloride, cystatin from chicken egg white and *N*-ethyl-*N*′-(3-dimethylaminopropyl)carbodiimide (EDC) were purchased from SIGMA, Steinheim, Germany. Human cystatin C and cathepsin D from the human liver were purchased from Merck, Darmstadt, Germany. *N*-hydroxysuccinimide (NHS), Tween-20, *N*-(2-hydroxyethyl)piperazine-*N*′-(2-ethanesulforicacid) (HEPES), pepstatin A. acquired from ALDRICH, Munich, Germany. In POCh, Gliwice, Poland absolute ethanol, sodium hydroxide, sodium chloride and sodium carbonate were purchased. Phosphate Buffered Saline (PBS, pH = 7.2) came from BIOMED, Lublin, Poland. Cathepsin S and rat monoclonal antibody specific to cathepsin S were purchased from R&D Systems, USA. Buffers were also used for the study: acetic buffer pH = 3. 75 and pH = 6.00, carbonate buffer pH = 8.50, HBS-ES pH = 7.40 (0.01 M HEPES, 0.15 M sodium chloride, 0.005% Tween-20, 3 mM EDTA). The gold-plated chips were purchased from SSens (http://www.ssens.nl/, accessed on 10 March 2022).

### 2.1. Biological Material

A total of 105 blood plasma samples were tested for each of the cathepsins, including 48 control samples, 3 at the G1 grade of advancement, 10 at grade G2, 7 at grade G3, and 37 at grade G4. All samples were obtained from the Biobank of the Medical University of Bialystok. The research obtained the consent of the relevant bioethics committee (permission APK.002.171.2021).

Table 1 shows patients’ clinical data.

**Figure A2:**
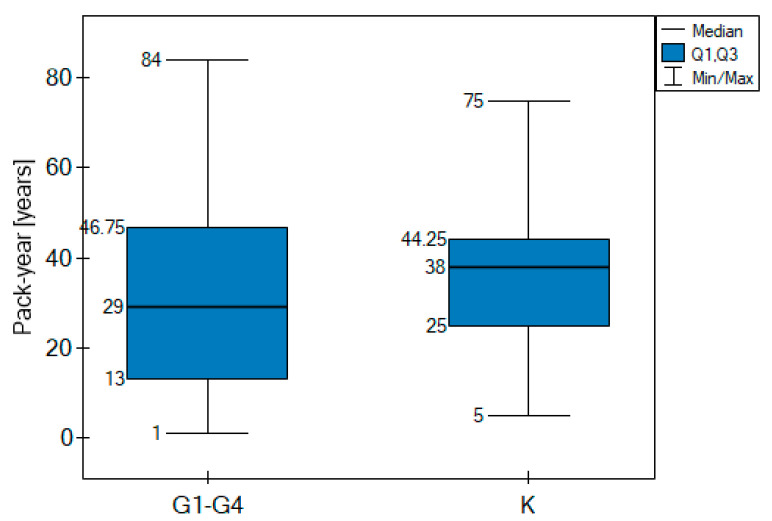
Analysis of the group of patients and the control group in terms of burned pack-years.

**Figure A3:**
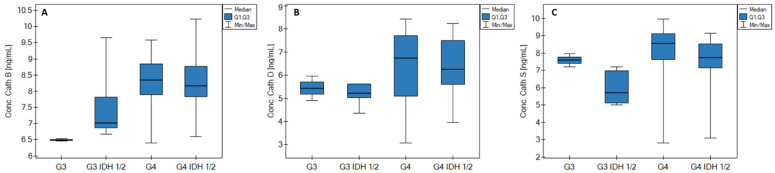
Effect of IDH status on concentrations (**A**) Cath B; (**B**) Cath D; (**C**) Cath S.

We examined whether the control group chosen for the research was not biased, that is, whether heavy cigarette smoking would falsify the results of the comparisons due to the possibility of slightly elevated concentrations of Cath B, Cath D and Cath S in this group. Statistical analysis ruled out such a possibility (*p* = 0.12, no statistically significant differences between the group of patients and the control group in relation to pack-years; Figure A2 in Appendix A). We also examined the effect of IDH 1/2 status on the concentrations of individual cathepsins in glioma grades G3 and G4. For this purpose, we performed the Kruskal–Wallis test and the Dunn–Bonferroni test as post hoc tests. We obtained data showing no effect of IDH 1/2 status on the determined cathepsins (*p* > 0.05 in each case and no statistical significance confirmed by the Dunn–Bonferroni test). We conclude from this that Cath B, Cath D and Cath S may be independent prognostic biomarkers of at least the grade of brain glioma. The results of the analysis in the form of a graph are presented in Figure A3 in the Appendix A.

### 2.2. Procedure for Quantifying Cath B, Cath D and Cath S

Quantitative determinations of Cath B, Cath D and Cath S were made using previously developed SPRi biosensors. Before the measurements, biosensors sensitive to individual proteases were prepared following previously described and published protocols. For the quantitative determination of Cath B, a biosensor was used in which cystatin C (C = 50 ng/mL) served as a ligand binding Cath B from the solution [24]. Cath D was determined with an SPRi biosensor in which the ligand was pepstatin A (C = 0.5 μg/mL) [25] while in the case of Cath S determinations, a rat monoclonal antibody was used as a ligand (C = 20 ng/mL) [26]. Blood samples were centrifuged for 15 min at 3000 rpm and then filtered three times. The blood plasma obtained in this way was stored at −80 °C. Because each of the cathepsins tested is stable in a different pH range, the plasma samples were diluted for each of them. For Cath B, plasma samples were brought to pH = 6.00, Cath D requires pH = 3.50, while Cath S has maximum catalytic activity at neutral pH (pH = 7.2). Blood plasma samples were diluted five times for G1 and G2, ten times for G3 and G4, and twice for the controls. This protocol was used for each of the proteases studied. The selectivity of the biosensors used was determined at the stage of validation of each of them and expressed in terms of recovery. The recovery values for each method are usually 100–104%, which means that the biosensors used for quantitative determinations in this study are selective methods.

### 2.3. SPRi Measurements

Surface Plasmon Resonance in the imaging version is a label-free optical technique, extremely sensitive to changes in mass occurring on the metallic surface, which are manifested by changes in the reflectance of light before and after the ligand–analyte interaction [27,28]. The device used for the research is based on the Kretschmann configuration and works in what is called stationary mode, that is, successive solutions are not brought to the surface of the biosensor by flow, but by applying drops (3 μL) to the active site of the biosensor. The device consists of, among other components, a light source (laser diode, λ = 635 nm), polarizer and lens systems, a glass prism made of BK-7 glass, and a biosensor placed on it. The detector is a CCD camera (resolution 1.4 MP), which collects a reflected beam of monochromatic light from the surface of the biosensor and converts it into an image. Measurements are carried out at one optimal angle of SPRi; hence, the first stage of research is to find the value of this angle based on a series of photos taken while an immobilized layer of binding ligand is present on the surface of the biosensor. The previously prepared blood plasma samples are then condensed and left for about 10 min to ensure interaction between the ligand and the analyte. After this time, the active sites of the biosensor are washed with MiliQ water and HBS-ES buffer to remove unbound particles from the biosensor surface. ImageJ 1.32 (National Institutes of Health, NIH) software converted numeric signals into quantitative signals. The concentrations of the proteases tested were derived from the equations of the calibration curves assigned to each of the analytical methods and the dilution used for the test.

### 2.4. IDH 1/2 Mutation, p53 Gene Mutation and EGFR Expression

The data on the status of molecular markers was taken from the available medical documentation describing the tested samples.

### 2.5. Statistical Analysis

Statistical analysis of the results was carried out using PQStat v.1.8.2 from PQStat Software (2021), Poznan, Poland.

**Figure 1:**
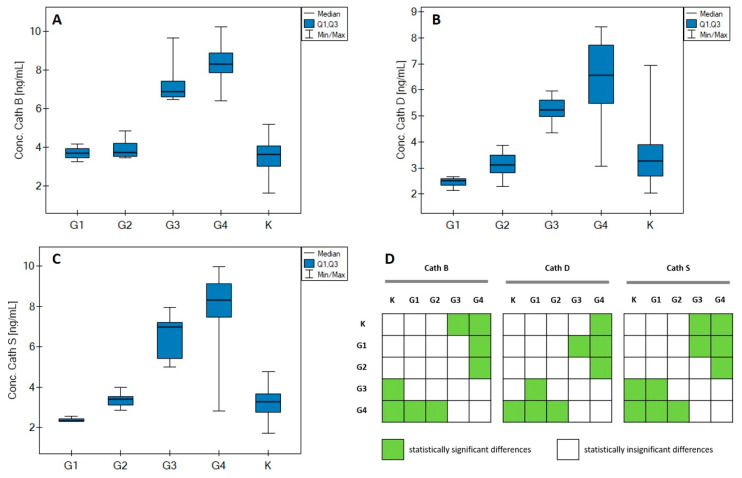
Changes in test concentrations of cathepsin: (**A**) cathepsin B, (**B**) cathepsin D, (**C**) cathepsin S, depending on the stage and in the control group. (**D**) graphical representation of Dunn Bonferroni’s test results.

The first step in the statistical analysis of the measurement results was to check the null hypotheses (H_0_) concerning the normal distribution of the results for concentrations of individual cathepsins. For this purpose, the Shapiro–Wilk normality test was performed. The value of the obtained statistic gave a statistically significant result, which led to the rejection of H_0_ and adoption of the alternative hypothesis (H_I_) of the lack of conformance of the results to the normal distribution. Further analyses were therefore performed using nonparametric tests, because, in addition to the lack of normality of the distribution of results, other conditions were detected favoring the use of this type of analysis; these concern the lack of equality of variance and the number of results in the studied groups. With this in mind, we performed the Kruskal–Wallis test, which is the nonparametric equivalent of univariate analysis of variance (ANOVA). For this test, H_0_ was taken to assert the absence of significant differences between the concentrations of each of the cathepsins studied depending on the stage of the disease, while H_I_ asserts that not all concentrations are equal. As a result of the analyses, a statistically significant test result (*p* << 0.01) was obtained, which allows the adoption of H_I_. Figure 1A–C shows the changes in the concentrations of the cathepsins studied in individual groups. Entries are presented as the median ± the first and third quartiles. The minimum and maximum concentration values of individual cathepsins are also noted (Table A1 in Appendix A).

To refine the data, we performed the Dunn–Bonferroni test, which should enable the selection of groups that show statistically significant differences and groups that do not have such a relationship. The results of the analysis are presented in the form of a graphical matrix in Figure 1D.

## 3. Results

### 3.1. Statistical Analysis

Significant differences between the concentrations are most often found between the control group and the higher grades of the disease (G3 and G4). Statistical differences between the mild G1 stage and the more aggressive stages are slightly less frequently detected.

The graphs show that cathepsin concentrations in individual grades remain at a similar level: for groups Control, G1 and G2, C = 1.62–6.95 ng/mL, while for G3 and G4, C = 2.81–10.23 ng/mL. We do not observe any major differences between the concentrations in groups Control and G1/G2. Differences occur between Control and advanced grades (G3/G4) and between milder grades (G1/G2) and more aggressive grades (G3/G4). In Table 2, a detailed characterization of changes in the concentrations of the studied cathepsins in relation to selected clinical data of patients is given.

We also correlated the concentrations of the tested cathepsins with the patient’s age. The Spearman rank correlation was used. Detailed data is presented in Table A2 in Appendix A.

### 3.2. Correlations

The *R_S_* correlation coefficients for the cathepsins examined are in the range $0.3<R_{S}\leq 0.5$ and exhibit statistical significance (*p* < 0.05). The value of the correlation coefficient indicates the average correlation of the concentration of a given cathepsin with the patient’s age. The data and conclusions relate to the statistical examination of all samples simultaneously. No relationships were seen when the samples were divided into individual glioma grades. This was probably due to the negligible number of G1 and G3 samples and the significantly larger number of G4 samples compared to all others.

We also correlated the quantities of the cathepsins with the size of the cancerous tumor (tumor surface area) and recalculated the values of Spearman’s rank correlation coefficients. In the case of cathepsin B and S, we observed a slight negative correlation between the amount of cathepsin and the surface area of the cancerous tumor. For cathepsin D, the correlation was positive, but also weak. All values of correlation coefficients were determined without obtaining statistical significance. This means that changes in the amounts of the studied cathepsins depending on the surface area of the cancerous tumor are accidental. As before, the above considerations apply to the analysis of all samples simultaneously.

The situation is slightly different in the case of mutual correlations between the studied proteins. A very high positive correlation (0.7 < *R_S_* ≤ 0.9) in the G2 grade was observed between Cath S and Cath D and between Cath B and Cath D. The result of these correlations may therefore have a diagnostic value for predicting the grade of G2 glioma. No dependencies were observed in the remaining grades. By re-analyzing all samples, a high positive correlation between proteins was proved (0.5 < *R_S_* ≤ 0.7). Detailed data are presented in Table A3 in the Appendix A. The strength of all correlations was assessed based on the J. Guilford scale.

### 3.3. ROC Analysis

**Figure 2:**
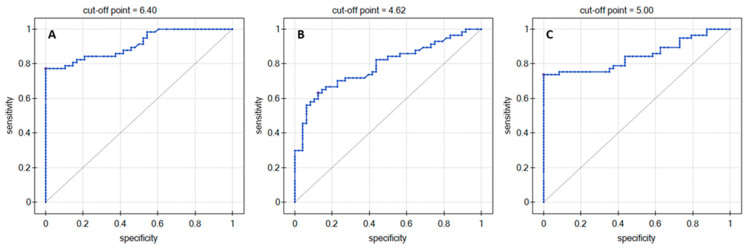
ROC curves as plasma diagnostic tests for (**A**) cathepsin B, (**B**) cathepsin D, (**C**) cathepsin S.

ROC analysis was performed using the DeLong method, comparing the control group dataset with the G1–G4 group dataset, without division into individual groups. Analysis of the ROC curves (Figure 2) shows that the concentrations of the individual cathepsins studied produced a quite clear distinction between patients with brain glioma (G1–G4) and the control group. The results of the ROC curve analysis for the cut-off points of each of the cathepsins are presented in Table 3. These include areas under the curve (AUC) along with the *p*-value, positive predictive value (PPV), and negative predictive value (NPV). Sensitivity, specificity and cut-off points for individual cathepsins are also included.

## 4. Discussion and Conclusions

The development of minimally invasive methods for the early diagnosis of brain tumors is extremely important, with regard to the possibility of rapid initial diagnosis as well as the physical and mental comfort of the patient. This work focused on the quantitative determination of cathepsins B, D and S in the blood plasma of patients with known brain glioma at various grades. The results were compared with those of a control group. This study represents the first attempt to determine the concentrations of these cathepsins in the case of brain glioma, their mutual correlation, and the correlation of the quantities of the studied cathepsins with selected parameters available in the clinical description of plasma samples.

For each of the cathepsins studied, statistically significant differences between groups Control to G4 were observed in the overall relationship. Thus, a Dunn-Bonferroni test was performed, revealing the data sets that exhibit and do not exhibit differences at a statistically significant level. The results of the test are shown in Figure 1D. The quantified cathepsins had similar concentration levels in groups Control, G1 and G2 (C = 1.62–6.95 ng/mL), and also in groups G3 and G4 (C = 2.81–10.23 ng/mL). In addition, no differences in the quantities of individual cathepsins were observed between group Control and groups G1 and G2. Statistically significant differences were found between group Control and groups G3 and G4, as well as between groups G1/G2 and G3/G4. The reason for this is probably related to the blood–brain barrier, the task of which is to prevent substances from entering the brain from the bloodstream. Brain tumors most often contribute to disorders of the function of the blood–brain barrier. The rapid growth of their cells causes the formation of areas of local hypoxia, which in turn activates the process of angiogenesis. In addition, in the structures of the blood–brain barrier, there are changes in the expression of proteins, more precisely aquaporins. All of this leads to the formation of an abnormal, pathological structure, which lacks the specific ability to perform the function of a barrier. The effect is strongest in the case of gliomas with a high grade of malignancy, where there is practically no longer any structure acting as a barrier between the bloodstream and the brain. However, it should also be remembered that all gliomas, even those with the highest grade of malignancy, exhibit local areas where the protective barrier of the brain has not been destroyed [5]. The statistical analyses performed proved the correctness of the selection of groups (sick and healthy) in terms of age and sex, and also refuted concerns about the bias of the control group, which consisted of heavy smokers (Table 1). This study did not demonstrate that either the presence of cancer in the patient’s immediate family or other comorbidities (other than cancer) increased the risk of brain glioma. Perhaps the main reason is the size of the study group (57 samples from glioblastoma patients) or the high uniformity of the samples at our disposal in terms of medical history. It was also not shown that IDH 1/2 status had an effect on the concentrations of the assayed cathepsins in glioma grades G3 and G4. This information may indicate that these cathepsins are independent prognostic biomarkers of the glioma grade. The correlation of Spearman ranks, according to the J. Guilford scale, proved the average correlation of concentrations of individual cathepsins with the age of patients. A similar analysis was performed between the amounts of particular cathepsins and the tumor size, giving values of correlation coefficients that indicated a slight dependence. The data obtained lacked statistical significance, which leads to the conclusion that the changes in the quantities of cathepsins depending on the surface area of the cancerous tumor are completely random. The correlations between individual proteins are much stronger. A very high positive correlation was found between Cath S and Cath D and between Cath B and Cath D in glioma grade G2. No similar relationships were found in the remaining grades. The correlations reappear when all samples are analyzed, suggesting a high positive correlation. The ROC analysis revealed a clear distinction between patients with glioma in grades G1–G4 and the control group. Table 3 summarizes the most important parameters of the ROC analysis of individual cathepsins. The presented values testify to the good specificity and sensitivity of the tested potential markers of glioma quantified in plasma.

According to the available literature data, elevated levels of cathepsin B, D and S are observed in various diseases, including cancer, among which gliomas at different grades of advancement are also mentioned [29]. The levels of obtained concentrations of cathepsins B, D and S presented in this work confirm the theories contained in the available literature. These data are also the first to be obtained from the plasma of patients with known brain glioma. However, due to the presence of a blood–brain barrier that protects against the entry of undesirable substances from the bloodstream into the brain, quantitative determinations capable of confirming or excluding the occurrence of glioma in the case of the tested potential biomarkers in the lower stages of the disease (G1–G2) are difficult to achieve. The concentration levels of the tested cathepsins at these grades do not differ significantly from the levels obtained in the analysis of the control group. The situation is different if we compare the milder grades (G1–G2) with aggressive grades (G3–G4), or the control group with grades G3–G4. In advanced, aggressive and infiltrating forms of the disease, hypoxia is likely to occur as a result of the rapid and uncontrolled growth of a cancerous tumor. This condition triggers the angiogenesis process. There is a network of impaired blood vessels and a structure incapable of preventing the free exchange of substances between the brain and the bloodstream. Proteolytic enzymes, such as cathepsins, as well as other products derived from the breakdown of basement membranes and metabolites, can therefore freely enter the bloodstream from the brain and vice versa. Therefore, in the G3–G4 grades, there is observed a sharp increase in the concentrations of the tested enzymes, which, however, remain at a similar level, so that it is not possible to distinguish the disease in the G3 grade from the disease in the G4 grade based only on concentrations of cathepsin B, D, or S. Additionally, the presence of cancers other than glioma in the patient’s immediate family and concomitant non-cancerous diseases in the diagnosed patient did not produce any correlations or differences in the quantities of the examined cathepsins.

Taking into account all of these relationships, it can be concluded that the cathepsins studied are promising biomarkers that may support the diagnosis of glioma, although a significant increase in their amounts is observed mainly in advanced stages. The proposed methods might also be used to predict the glioma grade when other diagnostic methods give ambiguous and uncertain results. However, it should be borne in mind that glioma diagnostics based on neuroimaging is performed at similarly advanced grades. A liquid biopsy, carried out using the patient’s blood, as compared to the collection of cerebrospinal fluid, would probably save the patient’s suffering and stress, and minimize the risk of side effects and failures during the procedure.

The direct competitor of the SPR is ELISA. The advantages of the SPR over the ELISA, however, lie in the label-free and quick and direct detection of the analytes. The analysis using the SPR also does not require as many washing steps as is the case with the ELISA. This makes it possible to detect molecules with lower affinity for the receptor, which could be washed out in the ELISA test. Most SPR biosensors can detect molecules at ng/mL and nM or higher levels. There are also more and more methods based on SPR that enable analysis at the level of pg/mL and pM. SPR enables the analysis of a wide range of biomaterials, including serum, plasma, urine, stool, whole blood, cerebrospinal fluid, white and red blood cells, saliva, endometrial tissue, cervico-vaginal discharge, and ascites fluid [30].

Indeed, a disadvantage of the developed methodology for the diagnosis of brain glioma is that it cannot be used to differentiate the G1/G2 grades from the control group, but it may be useful for differentiating the milder grades (G1/G2) from the more advanced grades (G3/G4). The obtained results of statistical analyzes are influenced to some extent by the unequal number of individual samples and the fact that the tests were carried out in blood plasma. Perhaps an analysis of the cerebrospinal fluid would distinguish the various degrees of the disease, but would make the method more invasive due to the need for invasive biopsy. As previously mentioned, in low-grade glioma, the tested cathepsins may have difficulties in freely crossing the BBB, hence the slight differences between the concentrations in the control group and the G1/G2 grades. The developed method will not replace imaging methods for the diagnosis of brain glioma and the analysis of molecular markers. It is a proposal to extend and support the diagnosis of brain glioma, which is quite a challenge. Research is even being conducted to use artificial intelligence algorithms to analyze MRI images and diagnose and differentiate the degrees of brain glioma. These algorithms also either encounter difficulties in the automatic analysis of very complex and non-uniform images giving erroneous and biased results, or require operation by qualified personnel [31]. The diagnosis of brain glioma is still difficult and provides a wide field for developing new, effective and non-invasive diagnostic methods as well as the search for sensitive and specific biomarkers.

## Figures and Tables

**Table 1:** Clinicopathological characteristics of patients.

Control Group/Tumor Grade							
Variable		Control *n* = 48	G1 *n* = 3	G2 *n* = 10	G3 *n* = 7	G4 *n* = 37	Age Difference between Control and G1–G4 ^A^
Age [years]	range	39–66	29–43	30–57	38–72	33–77	No statistically significant difference (*p* > 0.05)
	median	61	39	43.5	45	60	
		61	58				
Gender	male	27	1	5	3	26	
	female	21	2	5	4	11	
Tumor size [cm^2^]	<15		2	2	3	10	
	>15		1	2	1	10	
The presence of other neo-plasms in the immediate family	yes		0	6	3	17	
	no		3	4	4	20	
Concomitant non-cancerous diseases	yes		2	3	3	21	
	no		1	7	4	16	
Histological type of tumors	oligodendroglial		0	1	3	0	
	astrocytic		3	9	4	37	
Number of smokers	Number of cases	48	1	6	4	22	Pack-years difference between control and G1–G4 ^B^
	Pack-year (median)	38	0 ^C^	8	26	38.5	No statistically significant difference (*p* > 0.05)
		38	29				
IDH 1/2 mutation	yes		0	10	5	10	
	no		3	0	2	27	
p53 mutation	yes		1	10	7	34	
	no		2	0	0	3	
EGFR expression	yes		0	6	2	29	
	no		3	4	5	8	

^A, B^ U-Mann–Whitney test. ^C^ one case was 21 pack-years.

**Table 2:** Characterization of changes in cathepsin concentrations in relation to clinical data.

Parameter	Cath B Concentration [ng/mL]			Cath D Concentration [ng/mL]			Cath S Concentration [ng/mL]		
	Range	Median	*p*-Value	Range	Median	*p*-Value	Range	Median	*p*-Value
Tumor grade (G1–G2 vs. G3–G4)									
G1 (3)	3.25–4.17	3.68	<<0.01	2.13–2.66	2.51	<<0.01	2.30–2.54	2.32	<<0.01
G2 (10)	3.44–4.83	3.72		2.27–3.88	3.11		2.86–3.98	3.40	
G3 (7)	6.46–9.66	6.87		4.36–5.97	5.22		5.00–7.97	6.98	
G4 (37)	6.40–10.23	8.31		3.07–8.43	6.56		2.81–9.97	8.32	
Tumor size [cm^2^]									
<15 (17)	3.25–9.66	8.02	0.835 (NS)	2.51–8.36	5.62	0.677 (NS)	2.32–9.97	7.97	0.427 (NS)
>15 (14)	3.45–9.43	7.91		2.66–8.07	5.56		2.54–9.65	7.45	
The presence of other neoplasms in the family									
YES (26)	3.45–10.23	7.91	0.949 (NS)	2.94–7.72	5.49	0.563 (NS)	2.81–9.61	7.22	0.955 (NS)
NO (29)	3.25–9.66	7.75		2.13–8.43	5.98		2.30–9.97	7.54	
Concomitant non-cancerous diseases									
YES (29)	3.25–9.58	7.83	0.805 (NS)	2.27–8.43	5.79	0.643 (NS)	2.32–9.97	7.64	0.420 (NS)
NO (28)	3.44–10.23	7.91		2.13–8.07	5.56		2.30–9.65	7.22	

NS—no statistically significant values.

**Table 3:** Diagnostic efficacy of cathepsin B, D and S in plasma.

	AUC	*p*-Value	PPV [%]	NPV [%]	Sensitivity [%]	Specificity [%]	Cut-Off Point
Cath B	0.91	<0.001	100.0	78.7	77.2	100	6.40
Cath D	0.79	<0.001	85.7	66.7	63.2	87.5	4.62
Cath S	0.85	<0.001	100.0	76.2	73.7	100	5.00

**Figure A1:**
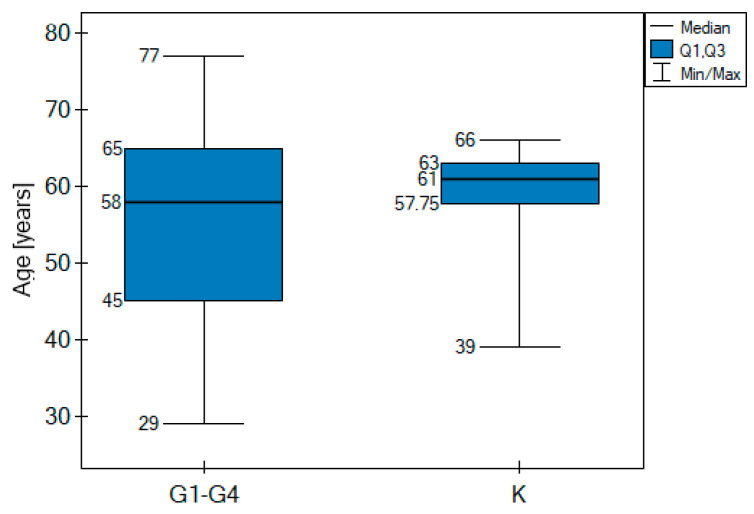
Age-related analysis of the patient group and the control group.

## Data Availability

Not applicable.

## Appendix A

**Table A1:** Detailed data for Figure 1.

Parameter/Biomarker	Cath B [ng/mL]					Cath D [ng/mL]					Cath S [ng/mL]				
	Control	G1	G2	G3	G4	Control	G1	G2	G3	G4	Control	G1	G2	G3	G4
**Median**	3.62	3.68	3.72	6.87	8.31	3.27	2.51	3.11	5.22	6.56	3.26	2.32	3.40	6.98	8.32
**Q1**	3.00	3.47	3.52	6.60	7.85	2.69	2.32	2.82	4.97	5.49	2.76	2.31	3.09	5.42	7.45
**Q3**	4.05	3.93	4.19	7.42	8.88	3.90	2.59	3.50	5.62	7.72	3.66	2.43	3.54	7.22	9.12
**MIN**	1.62	3.25	3.44	6.46	6.40	2.03	2.13	2.27	4.36	3.07	1.71	2.30	2.86	5.00	2.81
**MAX**	5.18	4.17	4.83	9.66	10.23	6.95	2.66	3.88	5.97	8.43	4.78	2.54	3.98	7.97	9.97

**Table A2:** Correlations: protein concentration–patient’s age and protein concentration–tumor size.

**Age–Cath B**					
	**G1**	**G2**	**G3**	**G4**	**All samples**
**R_Spearman_**	0.5	−0.27	−0.14	0.05	0.44
**p**	>0.05	>0.05	>0.05	>0.05	<0.01
**Age–Cath D**					
	**G1**	**G2**	**G3**	**G4**	**All samples**
**R_Spearman_**	-	0.06	0.46	−0.03	0.44
**p**	-	>0.05	>0.05	>0.05	<0.05
**Age–Cath S**					
	**G1**	**G2**	**G3**	**G4**	**All samples**
**R_Spearman_**	-	−0.34	0.05	−0.03	0.41
**p**	-	>0.05	>0.05	>0.05	<0.01
**Tumor size–Cath B**					
	**G1**	**G2**	**G3**	**G4**	**All samples**
**R_Spearman_**	-	−0.4	−0.2	−0.06	−0.11
**p**	-	>0.05	>0.05	>0.05	>0.05
**Tumor size–Cath D**					
	**G1**	**G2**	**G3**	**G4**	**All samples**
**R_Spearman_**	-	-	-	0.02	−0.04
**p**	-	-	-	>0.05	>0.05
**Tumor size–Cath S**					
	**G1**	**G2**	**G3**	**G4**	**All samples**
**R_Spearman_**	-	−0.8	0.2	−0.29	−0.18
**p**	-	>0.05	>0.05	>0.05	>0.05

**Table A3:** Correlation analysis between proteins.

**G1**			
	**Cath S–Cath B**	**Cath S–Cath D**	**Cath B–Cath D**
**R_Spearman_**	−0.5	-	−0.5
**p**	>0.05	-	>0.5
**G2**			
	**Cath S–Cath B**	**Cath S–Cath D**	**Cath B–Cath D**
**R_Spearman_**	0.55	−0.72	−0.71
**p**	>0.05	<0.01	<0.05
**G3**			
	**Cath S–Cath B**	**Cath S–Cath D**	**Cath B–Cath D**
**R_Spearman_**	−0.61	−0.35	0.03
**p**	>0.05	>0.05	>0.05
**G4**			
	**Cath S–Cath B**	**Cath S–Cath D**	**Cath B–Cath D**
**R_Spearman_**	−0.13	0.25	−0.07
**p**	>0.05	>0.05	>0.05
**All samples**			
	**Cath S–Cath B**	**Cath S–Cath D**	**Cath B–Cath D**
**R_Spearman_**	0.54	0.66	0.54
**p**	<<0.01	<<0.01	<<0.01
